# Surveillance system for Healthcare-associated endophthalmitis at state level in a middle-income country: preliminary results

**DOI:** 10.5935/0004-2749.2022-0181

**Published:** 2022-10-19

**Authors:** Reginaldo Adalberto Luz, Denise Brandão de Assis, Geraldine Madalosso, Stephen Timmons, Maria Clara Padoveze

**Affiliations:** 1 Faculdade de Ciências Médicas, Santa Casa de São Paulo, São Paulo, SP, Brazil; 2 Centro de Vigilância Epidemiológica, Departamento Estadual de Saúde, São Paulo, SP, Brazil; 3 Nottingham University Business School, Nottingham, UK; 4 Escola de Enfermagem, Universidade de São Paulo, São Paulo, SP, Brazil

**Keywords:** Epidemiological monitoring, Endophthalmitis, Delivery of healthcare, Health surveys, Ophthalmologic surgical procedures, Monitoramento epidemiológico, Endoftalmite, Atenção à saúde, Inquéritos epidemiológicos, Procedimentos cirúrgicos oftalmológicos

## Abstract

**Purpose:**

To describe the implementation pro cess and the preliminary results of a surveillance system for healthcare-associated endophthalmitis.

**Methods:**

This is a case study of the implementation of a surveillance system for healthcare-associated endophthalmitis. The system for healthcare-associated endophthalmitis is a structured system that enables surveillance of cases of healthcare-associated endophthalmitis after intraocular procedures, developed and coordinated by the Division of Hospital Infection at the State Health Department, São Paulo, Brazil. The implementation process included a pilot phase, followed by a scaling-up phase. Data were reported monthly to the Division of Hospital Infection by participating healthcare facilities that performed intraocular procedures in the state of São Paulo, Brazil, from September 2017 to December 2019.

**Results:**

Among the 1,483 eligible healthcare facilities, 175 engaged in the study (participation rate of 11.8%), reporting 222,728 intraocular procedures performed, of which 164,207 were cataract surgery and 58,521 were intravitreal injections. The overall incidence rate of endophthalmitis was reported to be 0.05% (n=105; 80 cases after cataract surgery and 25 cases after intravitreal injections). The incidence rates for healthcare facilities ranged from 0.02% to 4.55%. Most cases were caused by gram-positive bacteria, mainly *Staphylococcus* spp. In 36 (46.2%) of the cases, there was no bacterial growth; no sample was collected in 28 (26.7%) cases. This system for healthcare-associated endophthalmitis enabled the identification of an outbreak of four cases of endophthalmitis after intravitreal injections.

**Conclusion:**

The system for healthcare-associated endophthalmitis proved to be operationally viable and efficient for monitoring cases of endophthalmitis at the state level.

## Abstract (pt)

**Objetivo:**

Descrever o processo de implementação e os resultados preliminares de um sistema de vigilância epidemiológica para endoftalmites associada à assistência à saúde.

**Métodos:**

Trata-se de um estudo de caso de implementação de um sistema de vigilância epidemiológica para endoftalmites. O sistema de vigilância epidemiológica para endoftalmites é um sistema estruturado que possibilita a vigilância de casos de endoftalmite associados à assistência à saúde após procedimentos oftalmológicos invasivos, desenvolvido e coordenado pela Divisão de Infecção Hospitalar da Secretaria de Estado da Saúde, São Paulo, Brasil. O processo de implementação incluiu uma fase piloto, seguida pela fase de expansão. Os dados foram enviados mensalmente à Divisão de Infecção Hospitalar pelos estabelecimentos de saúde participantes que realizaram procedimentos oftalmológicos no estado de São Paulo, Brasil no período de setembro de 2017 a dezembro de 2019.

**Resultados:**

Entre os 1.483 estabelecimentos de saúde elegíveis, 175 participaram do estudo (taxa de adesão de 11,8%), relatando 222.728 procedimentos oftalmológicos realizados, sendo 164.207 cirurgias de catarata e 58.521 injeções intravítreas. A taxa de incidência global de endoftalmite relatada foi de 0,05% (n=105; 80 casos após cirurgia de catarata e 25 casos após injeção intravítrea). As taxas de incidência entre os estabelecimentos de saúde variaram de 0,02% a 4,55%. A maioria dos casos foi causada por bactérias gram-positivas, principalmente *Staphylococcus* spp. Em 36 (46,2%) casos não houve crescimento bacteriano; nenhuma amostra foi coletada em 28 (26,7%) casos. O sistema de vigilância epidemiológica para endoftalmites possibilitou a identificação de um surto de quatro casos de endoftalmite após injeção intravítrea.

**Conclusão:**

O sistema de vigilância epidemiológica para endoftalmites mostrou-se operacionalmente viável e eficiente para o monitoramento de casos de endoftalmite em nível estadual.

## INTRODUCTION

Intraocular procedures (IPs), such as cataract surgery and intravitreal injection of drugs for the treatment of age-related macular degeneration, are increasingly performed worldwide^(1^,^2)^. One of the possible complications of IPs is endophthalmitis, an intraocular infection that often leads to low visual acuity and, in many cases, to blindness^(3^,^4)^. The incidence rate of this type of infection can vary from 0.01%^(5^,^6)^ to greater than 0.12%^(7^, ^8^, ^9)^. Reports of outbreaks, which can affect many patients in a single day, are not rare^(10^,^11)^. Thus, the implementation of prevention strategies is essential to ensure the safety of patients undergoing IPs.

The World Health Organization considers surveillance one of the core components for infection prevention and control programs in health services^(12)^, contributing to driving preventive measures. The main objectives of surveillance are to know the magnitude of a problem and its etiology, identify populations at risk, and serve as a guide for public prevention policies^(12)^. In addition, surveillance systems enable the early detection of outbreaks and immediate implementation of control measures.

Post-IP surveillance systems are not common worldwide. Some countries, such as Sweden, Malaysia, Denmark, the United States, the United Kingdom, and France, systematically record IPs performed and their results, making it possible to obtain data about IP-related in-fections^(13^, ^14^, ^15^, ^16^, ^17^, ^18)^. Until 2017, there was no similar system in Brazil, thus hindering estimation of the incidence of post-IP endophthalmitis and the occurrence of outbreaks. The objective of this study was to describe the implementation process of the Surveillance System for Healthcare-Associated Endophthalmitis (SIVEN) and the main results obtained in the State of São Paulo, Brazil.

## METHODS

### Study design

This is an implementation study using a case study design.

### Study population and settings

The study was conducted in the state of São Paulo, which has more than 45 million inhabitants in 645 cities. Healthcare-associated infections (HAI) have been monitored in São Paulo since 2004^(19)^. To identify eligible healthcare facilities (HFs) (ophthalmology services), we consulted the database of the *Cadastro Nacional de Estabelecimento em Saúde* (CNES) in March 2016 and identified 1,483 eligible HFs.

### Surveillance System for Healthcare-Associated Endophthalmitis

SIVEN is a structured system that enables statewide gathering of essential epidemiological data on cases of endophthalmitis after IPs. This system is coordinated by the Division of Hospital Infection (DHI) of the São Paulo State Health Department. The Brazilian National Health System (*Sistema Único de Saúde*-SUS) ensures universal access to healthcare and is funded through tax collection; the participation of the private sector is complementary in the country. The system permits decentralized organization to the local level, and SIVEN was designed to follow the same principles. Information flows from the HF data to the health authorities of the municipalities, who in turn send information to the state surveillance structures distributed across 28 administrative regions called Surveillance Groups (SGs). SIVEN is structured to feature simplicity, taking into account the scarcity of both human and informatics resources in mostHFs. Monthly data are sent to SIVEN via email using Excel spreadsheets, containing pooled information or nonidentifiable individual information on patients with endophthalmitis. The following information is reported: number of IPs performed, number of cases of endo-phthalmitis detected and dates of diagnosis, identification and dates of the primary procedures (PPs) that progressed to endophthalmitis, number of days between the PP and diagnosis of endophthalmitis, and results of microbial culture of vitreous humor (when available).

### Case definition

The IPs selected to be monitored by SIVEN were cataract surgeries and intravitreal injections, considering their frequency in the Brazilian healthcare system. The definition of healthcare-associated endophthalmitis followed the criteria established by the Brazilian National Health Surveillance Agency (*Agência Nacional de Vigilância Sanitária* – ANVISA)^(20)^. The criteria were patient with isolation of microorganisms in microbiological culture of vitreous humor, OR patient who required intravitreal injection of antibiotics due to previous IP, OR patient with a diagnosis of endophthalmitis by the assistant physician. Cases were considered whenever the onset of signs/symptoms occurred up to 30 days after the PP in the absence of an implant, or 90 days in the presence of an implant.

### SIVEN implementation process

For the implementation of SIVEN, a guide was made available on the DHI webpage containing detailed information on the criteria for the case definition, methods of detecting cases, and how to report data. The implementation strategy had two phases. The first was a pilot phase from September 2017 to January 2019 with HFs from four selected SGs within the metropolitan region of São Paulo city. Participants were recruited by face-to-face meetings organized by DHI with representatives of the HFs, aiming to present SIVEN, its purpose, and its operational procedures. In February 2019, a feedback meeting with the participating HFs was held to present the preliminary results of the pilot phase. In addition, we aimed to identify possible barriers and facilitators encountered during the implementation of SIVEN, from the perspective of the participants. Participants were encouraged to express their opinions and suggestions, which helped to develop improvements and adaptations in SIVEN. In February 2019, the second phase (scaling-up) was implemented, aiming to expand SIVEN to all eligible HFs in the state of São Paulo. As part of the strategy to expand the implementation, all representatives of the SG and eligible HFs in the state participating in a web conference were invited to participate in SIVEN. Furthermore, societies of specialists in ophthalmology and representatives of teaching hospitals were considered as strategic stakeholders for this phase of implementation, and therefore specific meetings were held with them to present SIVEN, collect suggestions, and encourage participation. The data obtained by SIVEN were consolidated as pooled data to preserve the confidentiality of HF information and disseminated by the DHI to participating HFs through official bulletins.

### Data analysis

Statistical analysis of the data was performed using Epi Info™ version 7.2.5 (Centers for Disease Control and Prevention, Atlanta, GA). The adherence rate was calculated as the proportion of the number of participating HFs in relation to the number of eligible HFs.

### Ethical aspects

The study was conducted according to the principles of the Declaration of Helsinki^(24)^, being approved by the Ethics Committee of the institution where the work was undertaken, under the protocol number 68706317.9.0000.5392. Because this study was based on secondary data, the request for individual authorization was waived.

## RESULTS

The pilot phase started with 11 HFs and reached a maximum number of 33 HFs over 17 months. The scaling-up phase started with 51 HFs in February 2019, reaching a maximum number of 175 in December 2019. Among them, 97 HFs (55%) were private, 40 (23%) were public, and 38 (22%) were private not-for-profit.

**Figure 1:**
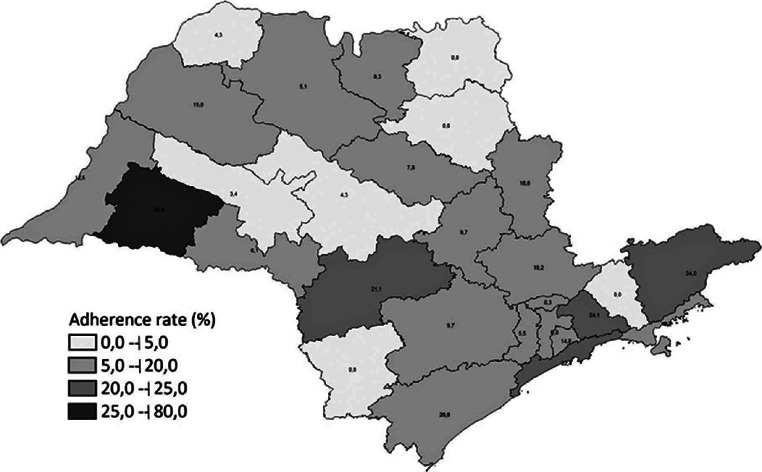
Adherence rates of healthcare facilities (HFs) to Surveillance System for Healthcare-Associated Endophthalmitis (SIVEN) by Epidemiological Surveillance Groups. São Paulo, Brazil, 2017–2019 (n=175).

The overall rate of adherence to SIVEN was 11.8% (175/1,483 HFs). The rate of adherence varied among the 28 SGs, with only one SG showing an adherence rate of 80% of HFs, and four SGs with no participation (Figure 1).

**Figure 2:**
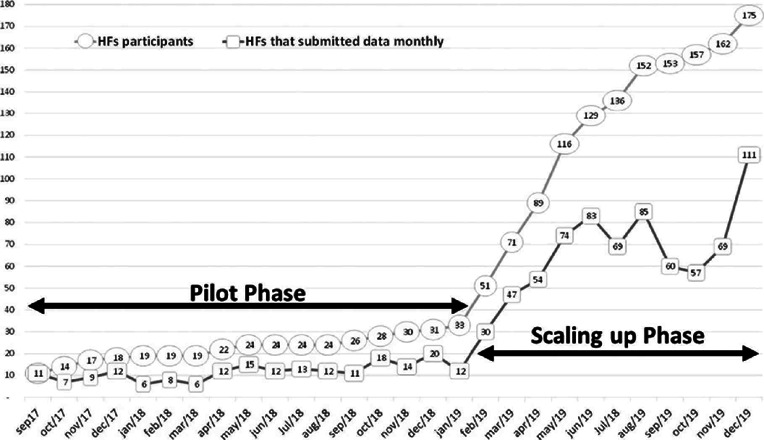
Monthly distribution of healthcare facilities (HFs) that were participants in Surveillance System for Healthcare-Associated Endophthalmitis (SIVEN) and those that submitted data monthly.

With regard to the regularity of data reporting, only 22 HFs (20%) submitted data for at least 80% of the period. SIVEN included HFs as participants from their first use of the system, noting that not all HFs entered the system at the same time (Figure 2).

During the study period (28 months), 222,728 IPs were performed by the HFs. Among these, 164,207 were cataract surgeries. The mean monthly number of cataract surgeries reported to SIVEN was 5,865 (ranging from 1,091 to 12,246; SD = 3,924). Although some HFs performed a large number of procedures, most HFs (n = 140/175; 80%) had an average of up to 100 cataract surgeries per month. The total number of intravitreal injections was 58,521, with a monthly mean of 2,090, ranging from 199 to 3,849 (SD = 1,226). Most HFs (n = 152/175; 86.9%) performed on the average fewer than 50 intravitreal injections per month (Table 1).

**Table 1:** Distribution of healthcare facilities (HFs) according to the monthly average number of intraocular procedures (cataract surgery or intravitreal injection) performed. São Paulo, Brazil, 2017–2019

Monthly average no. of intraocular procedures	Cataract surgery		Intravitreal injection	
	HFs*	(%)	HFs*	(%)
<10	34	(19.4)	120	(68.6)
10-49	66	(37.7)	32	(18.3)
50-99	40	(22.9)	9	(5.1)
100-199	25	(14.3)	10	(5.7)
200-499	6	(3.4)	3	(1.7)
500-1,000	3	(1.7)	1	(0.6)
>1,000	1	(0.6)	0	(0)
**Total**	**175**	**(100)**	**175**	**(100)**

* Number of HFs that perform the procedure.

A total of 105 cases of post-IP endophthalmitis were reported during the period of the study, for an overall incidence of 0.05%. Eighty cases of endophthalmitis were reported among the 164,207 cataract surgeries performed, for an incidence of 0.05%; 25 cases of endophthalmitis were reported among the 58,521 intravitreal injections performed, for an incidence of 0.04%. The incidence of endophthalmitis per HF ranged from 0.02% to 0.98%. Eight HFs reported an incidence less than 0.05%, 14 reported an incidence between 0.05% and 0.10%, and 16 reported an incidence greater than 0.11%. The highest incidence of endophthalmitis occurred in an HF that reported one case among 22 IPs performed during the period (Table 2).

**Table 2:** Number of intraocular procedures performed, reported number of endophthalmitis cases, and incidence rates of healthcare-associated endophthalmitis from the participants’ HFs. São Paulo, Brazil, 2017–2019

ID of HFs*	No. of intraocular procedures performed	No. of endophthalmitis cases	(%)
18	5,319	1	(0.02)
99	9,784	2	(0.02)
141	3,431	1	(0.03)
19	15,273	5	(0.03)
4	2,986	1	(0.03)
27	2,745	1	(0.04)
9§	21,756	8	(0.04)
93	2,643	1	(0.04)
120	2,154	1	(0.05)
10	1,923	1	(0.05)
26	1,825	1	(0.05)
24	1,816	1	(0.06)
49	1,801	1	(0.06)
105	1,669	1	(0.06)
34	1,658	1	(0.06)
162	1,492	1	(0.07)
21	1,483	1	(0.07)
48	2,666	2	(0.08)
11	1,110	1	(0.09)
132	1,063	1	(0.09)
170	1,008	1	(0.10)
8	11,942	12	(0.10)
116	849	1	(0.12)
51	1,507	2	(0.13)
144	729	1	(0.14)
42	7,211	10	(0.14)
160	3,956	6	(0.15)
16	7,418	12	(0.16)
87	605	1	(0.17)
88	941	2	(0.21)
114	1,093	3	(0.27)
7	353	1	(0.28)
25	2,217	7	(0.32)
52	272	1	(0.37)
29	643	3	(0.47)
106	369	2	(0.54)
96	613	6	(0.98)
61	22	1	(4.55)

* = Includes only HFs that reported at least one case of endophthalmitis.

§ = HF that reported an outbreak of four cases after intravitreal injection.

ID= Identification; HFs= healthcare facilities.

Among the participating HFs, 38 (21.7%) reported at least one case of endophthalmitis during the study period. Most HFs reported between one and three cases each, five HFs reported between four and nine cases, and three HFs reported between 10 and 12 cases.

The average number of days from the IP to diagnosis was 13.9 (range, 1 to 72; SD = 14.7); 76 (72.4%) of the cases were diagnosed within two weeks after the PP (Table 3). With regard to the microbiological profile of the etiological agents, 36 (46.2%) were caused by gram-positive bacteria, mainly *Staphylococcus* spp., which were identified in 24 (30.8%) samples. In 36 cases (46.2%), there was no bacterial growth; no sample was collected in 28 (26.7%) cases (Table 4). During the study period, an outbreak of endophthalmitis was reported, with four cases of endophthalmitis after intravitreal injections performed on a single day in the same HF. In this outbreak, the etiological agents (*Staphylococcus epidermidis* and *S. warneri*) were identified in two cases, but it was not possible to identify the etiological agent(s) in the other two cases. Notification of this outbreak through SIVEN acted as a trigger to start immediate actions by health authorities in support of the HF to control the outbreak. The actions included a review of the HF surgical work process to identify and correct gaps in the best practices for infection prevention.

**Table 3:** Number of days from the intraocular procedure to the date of diagnosis of endophthalmitis. São Paulo, Brazil, 2017–2019

No. of days	Cases related to cataract surgery		Cases related to intravitreal injection		Total cases	
	No.	(%)	No.	(%)	No.	(%)
1-4	17	(21)	11	(44)	28	(27)
5-15	36	(45)	12	(48)	48	(46)
16-30	14	(18)	2	(8)	16	(15)
>30	13	(16)	0	(0)	13	(12)
**Total**	**80**	**100**	**25**	**(100)**	**105**	**(100)**

**Table 4:** Microorganisms causing endophthalmitis after intraocular procedures. São Paulo, Brazil, 2017–2019 (n=77*)

Microorganism	No.	(%)
Coagulase-negative *Staphylococcus*	18	(23.1)
*Streptococcus* spp.	9	(11.5)
*Staphylococcus aureus*	6	(7.7)
*Enterococcus* spp.	3	(3.8)
*Haemophilus* spp.	2	(2.6)
*Proteus* spp.	2	(2.6)
*Acinetobacter* spp.	1	(1.3)
*Pseudomonas aeruginosa*	1	(1.3)
No growth	36	(46.2)
**Total**	**78**	**(100)**

*In one case, two microorganisms were identified.

## DISCUSSION

This study describes the experience and preliminary results of the implementation of a Surveillance System for Healthcare-Associated Endophthalmitis (SIVEN). The availability of the information about healthcare-related endophthalmitis allowed the magnitude of the problem to be recognized at the state level and favored benchmarking for the HFs, even for those who did not participate in the study. This is particularly important considering the large number of Brazilian HFs that perform IPs. Our experience may inspire the implementation of an analogous system in other states or at the national level, which had not yet been established at the time of development of this manuscript. Additionally, other countries in similar contexts can use our experience as a reference for implementing specific surveillance systems.

The adherence rate to SIVEN was low compared with studies that presented rates greater than 40%^(21^,^22)^; however, comparisons must be made with caution, since there are variations in the models adopted, resources available, and maturity of the surveillance systems.

One of the elements for evaluating the results of an implementation is the degree to which an intervention was implemented and executed according to what was planned^(23)^. In our study, a monthly regularity above 80% in sending of data by the HFs was expected, which did not occur. According to the Brazilian National Regulatory Agency (ANVISA), the ideal is for HAI surveillance data to be sent in at least 83% of the opportunities, that is, at least 10 times a year^(24)^. However, surveillance of healthcare-associated endophthalmitis is still an incipient activity in many HFs in Brazil and is frequently neglected when compared with the surveillance of other HAIs. The strategy used to implement SIVEN was adapted from that used during the implementation of the surveillance system for other HAIs in São Paulo, which was shown to be feasible and sustainable^(19)^. The adaptations were targeted to the specific features of HFs that perform IPs. We believe that the development of SIVEN may encourage the HFs to perform systematic monitoring of data on endophthalmitis, which requires a good organization of services for data collection and case identification, including the adoption of active searching for cases. During the implementation process, we observed that many HFs sent data with much delay, suggesting that there are flaws in the organization of services regarding data collection and analysis. This may hamper early detection of trends in increase of cases and outbreaks, reducing the prevention potential inherent in surveillance systems. The next step in the system will be promoting tailored interventions to improve adherence, data accuracy, and preventive measures, as in previous positive experiences in São Paulo^(25)^.

The overall incidence of endophthalmitis reported in the present study was similar to those reported in other countries^(1^,^26^,^27)^, but much lower than rates previously reported in other studies in Brazil, which were 0.13%^(3^,^7^,^28)^ or more^(9)^. There was great variability in this outcome among the participating HFs, which is also observed in the literature, in which rates vary from 0.01%^(5^,^6^,^29)^ up to greater than 0.12%^(7^, ^8^, ^9)^.

Our findings are similar to those of other studies concerning the time taken for the diagnosis of endo-phthalmitis after IPs, in which more than 70% of the cases were diagnosed within 15 days^(3^,^8)^, although in some situations, diagnosis occurs earlier, in up to four days^(3^,^9)^. This information indicates that surveillance systems can focus their efforts on active searches in the first two weeks after the procedure and thus optimize case capture.

The microbiological profile of the cases in which identification was possible was consistent with the world literature, in which agents found in the microbiota of the conjunctiva and human skin, such as *Staphylococcus* spp., are more frequently identified^(1^,^5^,^7^,^8)^. However, there was a low rate of positivity in the culture results, which has also been commonly reported internationally^(1^,^3)^. It is important to state that a negative microbial culture result cannot be used to rule out the diagnosis of endophthalmitis. Several factors can contribute to the absence of bacterial growth in intraocular content samples, such as the collection of insufficient material^(30)^ and the sample delivery time to the microbiology laboratory, which should be as short as possible^(31)^.

With regard to the outbreak of endophthalmitis after intravitreal injections detected in the data reported by one HF, it is not possible to say how much detection of the outbreak may have been favored by participation in SIVEN. Nevertheless, detection of outbreaks is a desired function of any surveillance system, and SIVEN may have helped to create awareness of endophthalmitis among participating HFs. As reported in other studies, outbreaks are common, since many ophthalmologic IPs are performed sequentially in a single day^(10^,^11)^.

Our study has some limitations. The system relies on accurate information from the HFs about the number of IPs, and a double-check process was not possible; however, experience with the surveillance systems of other HAIs does not indicate that major mistakes in the denominator are an issue^(19)^. On the other hand, the lack of detailed information on the process of case finding carried out by the participant HFs may limit full understanding of the problem. Therefore, participants with higher rates of endophthalmitis may not represent those with more failures in infection prevention, but rather those with better surveillance methods compared with other HFs with lower rates.

Preliminary data indicate that the implementation of SIVEN was feasible for large-scale monitoring of healthcare-related endophthalmitis. The data obtained by the system will support further initiatives for the prevention of endophthalmitis in São Paulo State, Brazil.
